# Narcissistic Personality Disorder: Are Psychodynamic Theories and the Alternative DSM-5 Model for Personality Disorders Finally Going to Meet?

**DOI:** 10.3389/fpsyg.2021.676733

**Published:** 2021-07-15

**Authors:** Frans Schalkwijk, Patrick Luyten, Theo Ingenhoven, Jack Dekker

**Affiliations:** ^1^Department of Forensic Special Education, University of Amsterdam, Amsterdam, Netherlands; ^2^Department of Clinical, Educational and Health Psychology, University College London, London, United Kingdom; ^3^KU Leuven, Leuven, Belgium; ^4^Arkin, Amsterdam, Netherlands; ^5^Department of Clinical Psychology, Vrije Universiteit Amsterdam, Amsterdam, Netherlands

**Keywords:** narcissistic personality disorder, alternative model for personality disorders, psychodynamic theory, hierarchical model for narcissism, intersubjective psychoanalysis

## Abstract

Narcissistic Personality Disorder is the new borderline personality disorder of our current era. There have been recent developments on narcissism that are certainly worthwhile examining. Firstly, relational and intersubjective psychoanalysts have been rethinking the underlying concepts of narcissism, focusing on the development of self and relations to others. Secondly, in the DSM-5, the Alternative DSM-5 Model for Personality Disorders (AMPD) was presented for a dimensional evaluation of the severity of personality disorder pathology. The combined dimensional and trait conceptualization of NPD opened the door to new integrated diagnostic perspectives, including both internal and interpersonal functioning. Finally, Pincus and Lukowitsky encourage clinicians to use a hierarchical model of pathological narcissism, as it opens up opportunities for shared points of interest in empirical research from different scholarly perspectives. As for most non-psychodynamic clinicians and researchers the DSM-5 clearly bears dominant weight in their work, we will take the AMPD model for NPD as our point of reference. We will discuss the narcissist's unique pattern of self-impairments in identity and self-direction, and of interpersonal disfunctioning (evaluated by assessing empathy and intimacy). Subsequently, we will examine how contemporary psychodynamic theories and the hierarchical model of Pincus and Lukowitsky additionally inform or contradict the AMPD. For us, one of the big advantages of the AMPD is the use of structured clinical evaluations of disturbances of the self and interpersonal functioning and the dimensional evaluation of severity. As psychodynamically oriented therapists, we are enthusiastic about the opportunities for inclusion of psychodynamic concepts, but we also discuss a number of sticking points.

## Introduction

Narcissistic Personality Disorder is the new borderline personality disorder of our current era (Choi-Kain, 2020). After three decades of progress have been made on Borderline Personality Disorder (BPD), Narcissistic Personality Disorder (NPD) now “… carries the potential for a new wave of investigation and treatment development.” Originally, narcissism was a psychoanalytic concept developed by Freud (1914). It became a dominant theme in the 1970s in the fierce debate between the psychoanalysts Kernberg (1975) and Kohut (1972). In the years that followed, few psychodynamic theoretical advances were made and research was scarce (as can be seen in Glasmann, 1988; Heiserman and Cook, 1998). However, in 1980, “given the increasing psychoanalytic literature and the isolation of narcissism as a personality factor in a variety of psychological studies,” narcissism found its way into the third Diagnostic and Statistical Manual of Mental Disorders (DSM-III; Frances, 1980, p. 1053). Narcissism had established a foothold in the diagnostic “bible.” In the decades since, a robust body of research has not developed to test or substantiate Frances' assumption that narcissism is a specific personality factor. In a recent online literature search on PubMed, Choi-Kain (2020) found 27 times more articles for BPD than for NPD. Even worse, research has found a significant overlap between the diagnostic criteria for all personality disorders in DSM-IV and extreme heterogeneity in patients with the same diagnosis (American Psychiatric Association, 2011). This conclusion was particularly clear in the case of NPD (Miller et al., 2010; Pincus, 2011). Not surprisingly, in the discussion preceding the publication of the DSM-5 (American Psychiatric Association, 2013), there was heated debate about radical changes to the criteria for personality disorder (Skodol et al., 2011; Oldham, 2015). Thirty years after the inclusion of NPD in the DSM-III, it was almost removed from the fifth edition.

However, in the past two decades, there have been developments relating to narcissism that certainly merit examination. Firstly, relational and intersubjective psychoanalysts have been rethinking the concepts underlying narcissism, focusing on the development of self and relations to others (Drozek, 2019). Secondly, an Alternative DSM-5 Model for Personality Disorders (AMPD) was established in the DSM-5 for the dimensional diagnosis of personality disorders alongside the strict categorical classification of personality disorders that had been used until then (Bender et al., 2011; American Psychiatric Association, 2013; Skodol et al., 2014a). In particular, the combined dimensional and trait conceptualization of NPD opened the door to new integrated diagnostic perspectives, including both internal and interpersonal functioning (Ronningstam, 2020a). Finally, Pincus and Lukowitsky's (2010) proposal for a hierarchical model of pathological narcissism opens up the prospect of looking beyond the relatively minor differences between competing theories about narcissism in order to find common ground.

In this article, we will examine if and how these recent developments can be integrated. We begin by providing an overview of contemporary psychodynamic theories on narcissism, followed by a description of the hierarchical model of narcissism and the AMPD for NPD.

## New Theoretical Developments

### Contemporary Psychodynamic Theories on Narcissism

An important question, clinically and conceptually, is what motivates human beings and makes them human. The traditional drive model posits that we are motivated by derivatives of innate aggression and sexual desires that can destabilize the ego or self. In recent decades, contemporary psychodynamic thinking has enriched conceptual knowledge about the motivational etiology and expression of narcissism. Turning away from the drive model implies relinquishing the assumption of specific narcissistic needs or a specific narcissistic phase in child development (Meissner, 2008). Instead, contemporary relational psychoanalysis focuses on attachment, mentalization, relational needs, and motivational affective systems (Modell, 1993; Panksepp, 1998; Akhtar, 1999; Meissner, 2009; Lichtenberg et al., 2011). As humans, we strive for development and homeostasis in self-organization, with biological and emotional forces playing an important role.

What shape does this take in optimal developmental circumstances? Self-organization develops with the adequate fulfillment of the emotional needs of babies and toddlers for attachment and emotion regulation (Schore, 2003). These needs are met in reciprocal interaction with significant others and represented in the brain as internal working models about the self, relations, and others (Bebee and Lachmann, 2002). In this development, the theory of object relations theory is also important. However, in the newer theories, the “relations” are based on a two-person psychology. These implicit working models are the materials for the “self-as-agent,” for sensing that you can prevent or make things happen. It is the blueprint for developing capacities for emotion regulation, attachment, mentalizing, reflective functioning, empathizing, and epistemic trust (Fonagy, 2003). As babies and toddlers have no capacity for speech and symbolic thinking, the self-as-agent remains implicit and can only be experienced by enacting it.

As the capacity for language and symbolizing increases, however, preschoolers arrive at the realization of the self as a subject that experiences emotion: the self-as-subject develops. The self-experience of a preschooler is relatively conscious as a person who gives meaning to his or her life and is separated from, while simultaneously attached to, significant others (Gergely and Unoka, 2008). Especially after the age of seven, the capacity for reasoning grows spectacularly and the child develops the capacity to self-reflect with a bird's eye view. Consequently, the self-as-object becomes integrated in a firmer sense of identity and the child constantly self-evaluates as in an inner dialogue (Meissner, 2008). The growing capacity for self-evaluation develops alongside the capacity to experience self-conscious emotions such as shame, pride, jealousy, and envy (Wurmser and Jarass, 2008; Schalkwijk, 2015, 2018).

We will now look at how this relational theory of self-organization can be applied to narcissism. The most important factor is the chronic frustration of the basic biological need for satisfying reciprocal interactions. A child's or toddler's frustration sets the scene for the development of dysfunctional capacities for emotion regulation, attachment, mentalizing capacities, reflective functioning, and empathizing. The self-as-agent feels more powerless than able to make things happen. Ronningstam (2020b) writes: “As a central aspect of narcissistic functioning, sense of agency influences both self-regulatory and interpersonal functioning, such as attention seeking, competitiveness, and achievements” (Ronningstam, 2020b, p. 91). These hampered capacities are part of the implicit self and thus operate outside of conscious awareness in the adult; they are ego-syntonic. Meissner (2008) and Symington (1993) suggest that, although not enacted “consciously” in the adult sense, the child has turned away from reciprocal interaction with others to protect his or her growing implicit self from chronic disappointment, from experiencing powerlessness instead of agency. Turning away from potentially frustrating interaction with significant others and opting for self-absorption is the core feature of pathological narcissism (Auerbach, 1993; Lachmann, 2007). This can already be observed in preschoolers. Brummelman et al. (2016) showed that preschoolers with a high score for either self-esteem or narcissism are differentiated by the latter verbalizing that they are great, others are stupid, interaction with others is frustrating, and one is better off on one's own. Those with high scores for self-esteem verbalized that they are great, others are great too, and working together will make the results better. This can also be seen in adult life. When one of our patients was persuaded by his children to play his computer games in the living room instead of sitting in the attic, he said: “I see no additional value in sitting downstairs. It is irritating as my daughters want me to get involved in what they are watching on TV.” Basically, the patient was unable to experience the pleasure of being with someone. Inevitably, by turning away from others, a frail self-as-subject results, as it is built on frustrating self and other representations that miss benevolent, soothing, and realistic qualities. As a result, self-regulation is further impaired as the development of the self-as-object is hampered as well. The capacity for self-knowledge through reflection on the subjective self is underdeveloped, protecting the subject from painful shame (Meissner, 2008). Consequently, in an unfortunate cumulation of hampered development, all aspects of the self are frail and self-regulation is dysfunctional.

Another relatively new psychodynamic theory, intersubjective psychoanalysis, has more to say about the dynamics of narcissism (Benjamin, 2018; Drozek, 2019). By contrast with the basic need for satisfying reciprocal interactions posited by relational psychoanalysis, intersubjective psychoanalysis stresses the intrapsychic motivation for the intention to relate. Imagine not only being motivated by biological needs but also being intrinsically motivated to relate (“just for the fun of it”). Imagine wishing to recreate being in a relationship with another and re-experiencing the fulfillment that gives. According to Benjamin (2018), this makes human beings fundamentally subjects who unconditionally value themselves and the other as individually dignified. Another fundamental characteristic of narcissism, in addition to incoherent self-organization, is a severe impairment of the intrinsic motivation to seek nearness and recognize the other as a subject.

In the next section, we will explore the trauma of narcissism and the associated suffering. Drozek (2019) states that patients with severe pathological narcissism (or borderline problems) find it impossible to value themselves unconditionally or ascribe unconditional value to others. They are therefore unable to be motivationally receptive to the subjectivity of others. “Rather, these patients are often only valuing aspects of the other (e.g., attentiveness, admiration, dependency) and valuing themselves only conditionally (e.g., contingent on their ability to appease the other)” (Drozek, 2019, p. 93). In this paper, we will not enter into the therapeutic implications of an intersubjective stance of this kind. We will go no further than pointing out that the therapist should actively assume responsibility for repairing ruptures in the relationship between the patient and the therapist (Benjamin, 2018). Recognition from the therapist is insufficient for change; patients should also be actively engaged in recognizing themselves and the therapist/others. Recognition implies owning one's vulnerability and harmful aspects instead of projecting them onto the other.

The lack of intrinsic motivation for relating is associated not only with psychological distancing from and only conditionally valuing others, but also with another recent theoretical focus, namely, attachment theory. Diagnostically, one would expect insecure attachment styles. The lack of intrinsic motivation for relating would then emerge in a dismissive-avoidant attachment style, whereas the extrinsic motivation for relating, as seen in excessive reference to others for self-enhancement, would be seen in a preoccupied attachment style. Research into the relationship between pathological narcissism and attachment styles is scarce but it is growing. Banai et al. (2005) suggest that the painful longing for others to fulfill one's own needs may be a motivational component of attachment avoidance: “I don't need you!” Exploring early life experiences in a non-clinical sample, Cater et al. (2011) showed that narcissistic dynamics like entitlement, grandiosity, and vulnerability were associated with different parenting styles. Summarizing the research findings to date, Diamond et al. (2013) conclude: “Narcissistic disorders have been associated with dismissing-avoidant attachment status (…) but patients may also be characterized by preoccupied attachment status, in which the individual remains angrily or passively enmeshed with attachment figures” (Diamond et al., 2013, p. 533; see also: Ronningstam, 2020b).

In the clinical and research literature, we see specific countertransference feelings in narcissistic patients as valuable contributions to the diagnostic process. In a clinical sample, independent of the therapist's theoretical orientation, age, or gender, NPD was positively associated with criticized/mistreated and disengaged countertransference, and negatively associated with a positive therapist response (Tanzilli et al., 2015, 2017). Further research in a sample of adolescents showed that grandiose narcissistic traits were associated with angry/criticizing and disengaged/hopeless therapist responses, whereas warm/attuned therapist responses fell short (Tanzilli and Gualco, 2020). In addition, the quality of the therapeutic alliance was lower. Adolescents with hypervigilant traits received overinvolved/worried therapist responses and few angry/criticized responses^1^.

These countertransference reactions may indicate a dismissive attachment style in the patient. The negative association with positive therapist response confirms our clinical experience. As a patient said: “When you are so kind to me, I want to hit you!” The therapist's kindness or benevolence evokes shame: the patient, who is in a help-seeking, dependent position, finds the therapist's kindness humiliating. Envy can be used as a defense against shame: the patient envies the therapist's superiority and wants to take it away from him or her (Morrison and Lansky, 2008). The dynamics between shame and envy express themselves in a self-focused competitive view of others that is considered to be a characteristic of narcissism. All relations here are thought to be about winning or losing, and mutual advantage is an unthinkable reality, as seen in the aforementioned research with preschoolers by Brummelman et al. (2016).

In this paper, we depart from this contemporary relational and intersubjective line of psychodynamic theorizing, with characteristics such as the loss of reciprocal interaction, the loss of intrinsic motivation for seeking nearness, ascribing only conditional value to oneself and others, frail self-regulation, and the absence of the self-as-object. More traditional psychodynamic theories will not be replaced or dismissed and will continue to be referred to when applicable. Throughout this paper we will also refer to the Psychodynamic Diagnostic Manual, Second Edition (PDM-2, Lingiardi and McWilliams, 2017). The PDM-2 focuses on personality styles and not on personality disorders. Personality styles are “a relatively stable confluence of temperament, attachment style, developmental concerns, defenses, affect patterns, motivational tendencies, cultural influences, gender and sexual expressions and other factors–irrespective of whether that personality style can be reasonably conceptualized as ‘disordered”' (McWilliams et al., 2018, p. 299). The term personality disorder is used for personality styles “denoting a degree of extremity or rigidity that causes significant disfunction, suffering, or impairment” (Lingiardi and McWilliams, 2017, p. 17). The PDM-2 is based on the integration of the vast body of clinical experience with the richness of empirical research, thus departing from the DSM-5's fundament of empirical research only. In contrast to the DSM-5's striving for simplicity by ascribing fixed patterns of symptoms, the fundamental psychoanalytic premise in the PDM-2 is that doing complexity justice by acknowledging that “opposite and conflicting tendencies can be found in everyone (McWilliams et al., 2018, p. 300).”

### The Hierarchical Model of Narcissism

**Figure 1:**
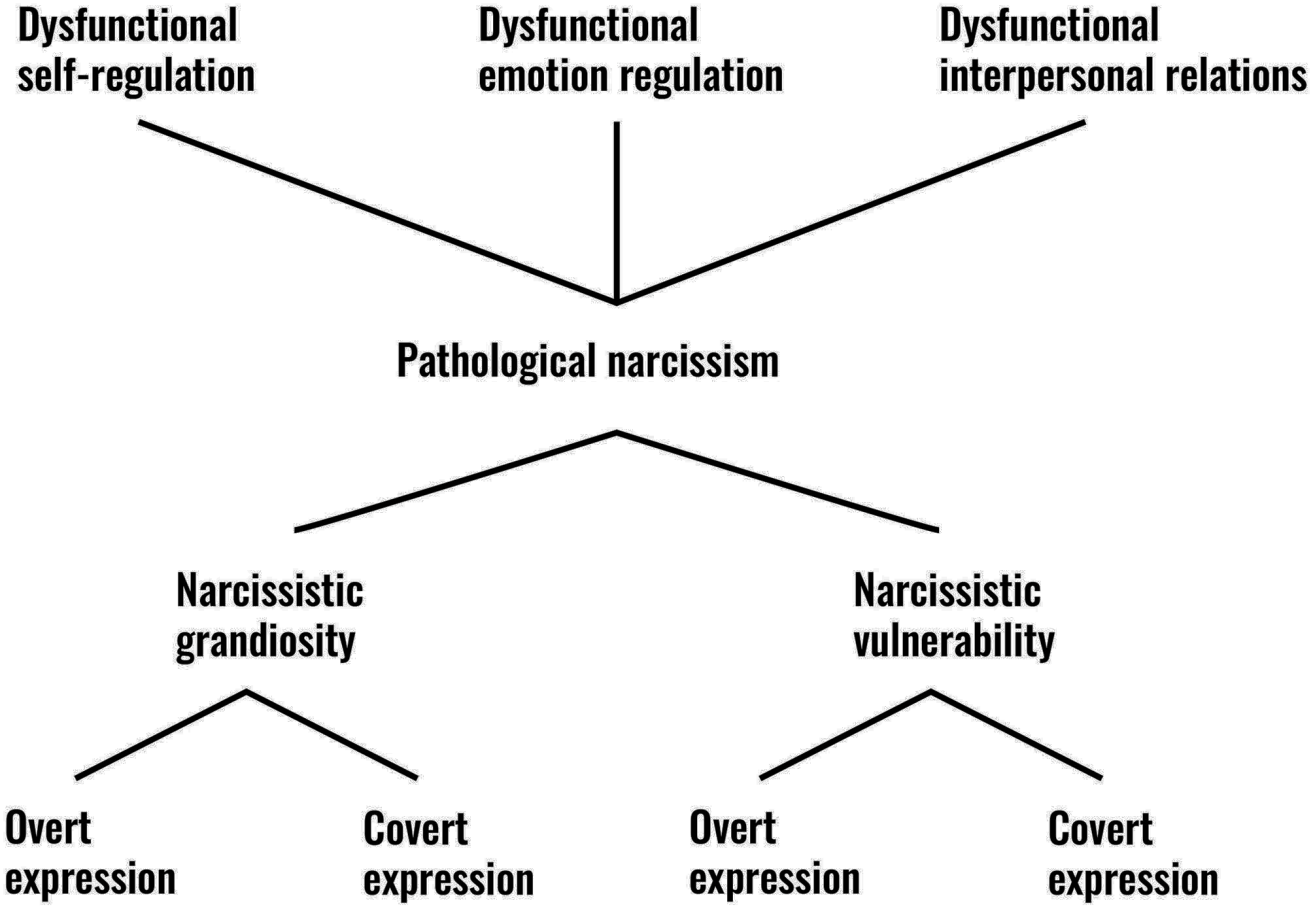
Pincus and Lukowitsky's model of narcissism.

Synthesizing theories about narcissism with the results from research and leaving the “narcissism of minor differences” behind, Pincus and Lukowitsky (2010) proposed that pathological narcissism is best conceptualized by a hierarchical model (see Figure 1). In their view, pathological narcissism is basically characterized by a combination of three psychodynamic phenomena: dysfunctional self-regulation, emotion regulation, and interpersonal relations.

They consider these three dysfunctional phenomena to represent the most basic building blocks of pathological narcissism. From this perspective, in contrast to the DSM-5 NPD classification, the Pincus and Lukowitsky model allows pathological narcissism to be situated on a continuum between two prototypes, which are covered by different terms in the clinical and research literature. At one end of the spectrum we find the prototype of grandiose, thick-skinned, arrogant/entitled, shameless, oblivious narcissism (PDM Task Force, 2006; Gabbard, 2015). At the other end, we see the prototype of vulnerable, thin-skinned, hypervigilant, shame-prone, depressed/depleted narcissism: “This *narcissistic vulnerability* is reflected in experiences of anger, envy, aggression, helplessness, emptiness, low self-esteem, shame, social avoidance, and even suicidality” (Pincus, 2013, p. 95; italics Pincus). Although empirical evidence is still lacking, Pincus and Lukowitsky assume that grandiose and vulnerable narcissism can express themselves both overtly and covertly. “Thus, we might diagnose a patient with grandiose narcissism, with some elements being expressed overtly (behaviors, expressed attitudes and emotions) and some remaining covert (cognitions, private fantasies, feelings, motives, needs)” (Pincus, 2013, p. 96).

An interesting line of research was adopted by Russ et al. (2008) with the Shedler-Westen Assessment Procedure. They used atheoretical Q-sort methodology to identify, in addition to those described by Pincus and Lukowitsky, two subtypes of narcissistic personality disorder, as well as a high-functioning/exhibitionistic subtype. Patients with this third subtype, who are well represented in the clinical literature, “have an exaggerated sense of self-importance, but are also articulate, energetic, and outgoing. They tend to show good adaptive functioning and use their narcissism as a motivation to succeed” (Russ et al., 2008, p. 1479). This third subtype could be the prototype of the positive side of narcissism, a line which has not received much attention.

In their model, therefore, pathological narcissism is basically characterized by a dysfunctional regulation of self, emotions, and relations, which is remarkably consistent with contemporary relational psychodynamic theorizing. Pathological narcissism can therefore be situated between the poles of grandiose and vulnerable narcissism, which is consistent with traditional psychoanalytic theorizing but not with the original NPD concept in DSM-III and later editions. The idea that narcissism can express itself overtly and covertly is consistent with traditional psychoanalytic theory.

### The Alternative Model for Personality Disorders

As stated above, the American Psychiatric Association (APA) discussion about the classification of personality disorders led to two different classification approaches in DSM-5. The first classifies the patient as usual in one of the official ten personality disorder categories, as described in section II of DSM-5. Clinicians and researchers can also adopt the new AMPD approach described in section III to assess patients' level of personality functioning and their unique trait profile. The assessment then consists of a mixture of clinical evaluation and the use of standardized instruments (Skodol et al., 2014b; Berghuis et al., 2017). In the AMPD, each personality disorder is characterized by a specific pattern of personality disfunctions and traits. In the case of narcissistic personality disorder, there is a unique pattern of self-impairment in identity and self-direction, and of impaired interpersonal functioning in empathy and intimacy. An NPD diagnosis is justified when at least two of these four elements are moderately or severely impaired. The specific traits to be assessed are grandiosity and attention seeking. It is interesting to note that, in PDM-2, the level of severity is established along the lines of Kernberg's concept of neurotic, borderline, and psychotic personality organization (Lingiardi and McWilliams, 2017).

In the next section, we will address the four AMPD elements of personality functioning and its specified traits on the basis of current psychodynamic concepts and the hierarchical model described above.

## Reflection on Personality Impairments in Narcissism

In order to integrate the recent developments discussed here, we need a point of reference. As is the case for most non-psychodynamic clinicians and researchers, DSM-5 clearly plays a role in our work, and so we will adopt the AMPD model for NPD as our point of reference. Subsequently, we will examine how contemporary psychodynamic theories and the hierarchical model of Pincus and Lukowitsky additionally inform or contradict the AMPD.

### Evaluating Impairment of Identity

The AMPD conceptualizes identity impairment as:

- excessive reference to others for self-definition and self-esteem regulation;
- exaggerated self-appraisal, inflated or deflated, or vacillating between extremes; and
- emotional regulation mirrors fluctuations in self-esteem (American Psychiatric Association, 2013, p. 776).

This conceptualization addresses the function of others for self-definition and self-esteem regulation. Reference to others for self-definition is adequately described in traditional psychodynamic theorizing. Kohut (1972) emphasizes how the patient uses others instrumentally as objects for enhancing the patient's self, calling them “self-objects.” As soon as others no longer fulfill that function, their instrumental value becomes zero, and they are devalued as losers and discarded. Although this could appear to be counterintuitive, we argue that this applies not only to grandiose, but also to vulnerable, narcissism. In the latter, the patient enhances self-esteem by placing others in the spotlight.

Another counterintuitive combination is the AMPD's stress on “excessive reference to others” and the psychodynamic view that narcissism implies a refusal of reciprocal interaction with others and a lack of intrinsic motivation for nearness. The key to bringing together these seemingly different foci lies in the answer to the question “excessive reference to which self and which others?” The implicit self is consciously verbalized as a subjective self on the lines of: “I do not want to think and talk about the distress of my partner; I cannot bear it. It is too threatening to myself.” The narcissistic patient refuses to recognize the unconditional value of the other and to live in a reciprocal world. Indeed, others do “excessively” matter but not as unconditionally valuable subjects: their relational value depends on the instrumental function they serve for the regulation of the patient's self-esteem. We agree with Meissner (2008), who sees narcissism as a psychodynamic function motivated by the need for “self-definition, self-development, self-organization, self-preservation, self-cohesion, self-enhancement, self-evaluation, self-regard, and self-esteem” (Meissner, 2008, p. 768). We are in favor of interpreting the strong focus on self-definition in AMPD's NPD as a focus on striving for coherence of identity. As for the quality of the excessive reference to others, we should not forget that, even if this reference becomes explicit, it is still located in the internal framework of a dysfunctional implicit self. Fonagy et al. (2002) add that the dysfunctioning of the self is further caused by the underdevelopment or absence of the self-as-object. Self-reflection and introspection are therefore impaired, and so is self-knowledge.

Identity is further conceptualized in the AMPD as “Self-appraisal inflated or deflated, or vacillating between extremes” and “Un-nuanced: self-loathing, self-aggrandizing, or an illogical, unrealistic combination” (American Psychiatric Association, 2013, p. 777). Likewise, in the PDM-2, the narcissistic personality style's central tension or preoccupation is inflation vs. deflation of self-esteem, whereas defense organization is dominated by idealization and devaluation (Lingiardi and McWilliams, 2017). Combining this definition with psychodynamic theorizing, we must differentiate between two diagnostic groups. In patients with narcissism, the subconscious dysfunctional regulation of the subjective self lies in its incoherence, in the vacillation between black-and-white opposites of idealization and devaluation. The patient is therefore engaged in a constant struggle with himself or herself; even narcissistic grandiosity co-occurs with insecure self-representations and sensitivity to rejection (Kealy et al., 2015). Caligor (2013) maintains that “as identity pathology becomes more severe, overt pathology in the sense of self as in the sense of others emerges” (Caligor, 2013, p. 71). In the other group who could fit this description, however, patients consciously suffer from low self-esteem. Their self is consciously experienced as consistently defective in only one direction: failing and coming up short.

Finally, the third element of identity impairment is “emotional regulation mirrors fluctuating self-esteem” (American Psychiatric Association, 2013, p. 777). In narcissism, emotions follow momentary self-esteem states whereas, in BPD, for example, self-esteem would appear to follow emotions more. One of our patients reported that her weekend had been depressing. She had frequently tried to help friends but, in the end, none of them had needed her. Where did that leave her? She felt useless and therefore depressed. The link between self-esteem and dysfunctional emotion regulation is characteristically expressed in the concept of narcissistic rage: the patient is extremely vulnerable to humiliation (perceived or otherwise) and strikes out when others are disappointing (Kohut, 1972). The PDM-2 focuses on shame, humiliation, contempt, and envy as central affects (Lingiardi and McWilliams, 2017). In a study of grandiose narcissism, shame was found to act as a mediating factor, reducing levels of aggression in patients with perfectionistic traits (Fjermestad-Noll et al., 2020). Clinically, this vulnerability is strengthened by the experience of shame when identity is negatively evaluated. Much more than guilt, shame is associated with falling short of one's expectations of an ideal, grandiose self. Shame is differentially associated with the aspect of grandiosity vs. vulnerability. Generally, shame is absent or warded off in grandiose narcissism, whereas grandiose fantasies can alternate with intense shame about needs and ambitions in vulnerable narcissism (Gramzow and Tangney, 1992; Dickinson and Pincus, 2003; Ronningstam, 2005). A more recent explanation for this fluctuation is that some patients with NPD tend toward mental concreteness, a refusal of symbolization or not symbolizing (Ronningstam, 2020b). This certainly has severe implications for the therapeutic alliance, the limitation of latitude for interpretation, and countertransference in the therapist.

### Evaluating Impairment of Self-Direction

The AMPD conceptualizes the impairment of self-direction as: “Goal setting based on gaining approval from others; personal standards unreasonably high in order to see oneself as exceptional, or too low based on a sense of entitlement while frequently unaware of one's own motivations” (American Psychiatric Association, 2013, p. 767). The PDM-2 also describes as characteristic the pathogenic belief about self that “I need to be perfect to feel OK,” whereas the pathogenic belief about others is: “Others enjoy riches, beauty, power, and fame; the more of those I have, the better I will feel” (Lingiardi and McWilliams, 2017).

With respect to the element of “goal setting based on gaining approval from others,” our clinical experience is that the patient can experience approval with no connection to reality. Consequently, others do not have to express their gratitude or approval in order to fulfill their instrumental function. In the splendid isolation of covert narcissism, admiring others can very well be imaginary: “Once I have published my solution for the global warming problem, everybody will admire me.” The internal (and possibly hidden) goal setting, which can take place in fantasy or daydreaming and with no footing in reality, is a particular inaptness in goal setting in covert narcissism that can be easily overlooked by clinicians.

The general inaptness of personal standards that is mentioned in the AMPD is clinically highly recognizable and consistent with psychodynamic theorizing. The suggested associations between “high standards and being exceptional” vs. “low standards and being entitled,” however, do not do justice to the converse clinical reality that high goal setting may also be based on the belief of being entitled and low goal setting on the belief of being exceptional anyway. Psychodynamic authors have provided good descriptions of the psychodynamics of shifting defenses in narcissism, in other words the warding of one emotion with another. For example, a patient can feel exceptional by setting extremely low standards, as in the patient mentioned above: “Once I have published my solution for the global warming problem, everybody will admire me. It's all in my mind, I just have to write it up when I feel it's time to do so.” Until then, the patient will just go on as usual, keeping a low profile.

Finally, AMPD and psychodynamic theorizing match up straightforwardly in the idea of being “often unaware of one's own motivations”: self-knowledge has to be avoided at any cost and often the patient has no conscious knowledge of struggling with his or her self-esteem or identity. We have already described the phenomenon in which the less patients can reflect upon themselves—an indication of weak reflective functioning—the more pathological narcissism is likely. To the best of our knowledge, little research has been conducted until now that specifically addresses the ability of reflective functioning in narcissistic patients (Diamond et al., 2013, Ronningstam, 2020b).

In our clinical experience, narcissistic patients live their lives and use treatment at their own pace: “Time is on my side.” This makes treatment targeting inner change extremely difficult and time-consuming. Making narcissistic dynamics egodystonic and sensitizing the patient to hidden motives is one thing but handling the high levels of shame and anxiety that accompany the uncovering of the implicit self, which the patient feels compelled to ward off, is another (Steiner, 2011).

### Evaluating Interpersonal Impairment in Empathy

With the discussion of empathy, we enter the world of interpersonal difficulties encountered by narcissistic patients. The AMPD conceptualizes empathy as the: “Impaired ability to recognize or identify with the feelings and needs of others; excessively attuned to reactions of others, but only if perceived as relevant to self; over- and underestimate of own effects on others” (American Psychiatric Association, 2013, p. 767).

The aspect of “impaired ability to recognize or identify with the feelings and needs of others” fits in well with Pincus and Lukowitsky's hierarchical model of pathological narcissism. In that model, impairment in interpersonal functioning is one of the three basic features of narcissism. Narcissism is accompanied by an impaired ability to identify the feelings and needs of others, the failure to recognize the other as a subject in her or his own right, and blocking reciprocity and mutual affect regulation (Ritter et al., 2011). The patient does not expect to benefit from sharing emotions and is not intrinsically motivated to seek nearness. The impairment in empathy is not only found in impaired mentalizing: as patients are not willing to focus their attention on the other, they will also not want to respond emotionally to what can be experienced through empathy (Allen et al., 2008). In clinical practice, the therapist's empathic interventions are often warded off by an *empathic wall*: “I don't want to be understood by you” (Nathanson, 1986).

The qualification of the patient as being “excessively attuned to reactions of others, but only perceived as relevant to self” is very apt. In as much as others do not threaten to destabilize the patient's self-esteem, they are not in the patient's mind. If empathy does come into play, the quality of empathy is most likely to be extremely poor as others are perceived on the basis of the patient's subconscious blueprint of the implicit self. In research literature on empathy, there is a distinction between affective and cognitive empathy, which are represented in two different neural circuits (Fonagy et al., 2002; Cuff et al., 2016). Clinically, if the patient has some empathic awareness of the other, we would expect cognitive empathy to be more associated with grandiose narcissism, and affective empathy to be more associated with vulnerable narcissism. Research, however, does not support our clinical experience: NPD patients have significant impairments in affective empathy, whereas cognitive empathy seems largely unaffected. Despite our clinical experience, Ronningstam (2020b, p. 84–85) concludes: “Further studies have provided evidence for compromised empathic function in NPD, that is, intact cognitive but neural-deficient emotional empathy, and impact of emotion intolerance and processing on ability to empathize (Ritter et al., 2011).”

### Evaluating Interpersonal Impairment in Intimacy

The AMPD conceptualizes intimacy as follows: “Relationships are largely superficial and exist to serve self-esteem regulation; mutually constrained by little interest in other's experiences and predominance of a need for personal gain” (American Psychiatric Association, 2013, p. 767). Relationships of this kind are related to the etiology of pathological narcissism represented in the blueprint of the implicit self: the inner representations of others are not based on an integration of differentiated images of self and others, nor are others recognized as autonomous subjects. Indeed, patients only send; they do not receive and they refuse reciprocity in relations with others. They hardly engage at all in inner self-talk as someone with a well-developed self-as-object would do to acquire more self-knowledge. It should be remembered that others are not seen as persons in their own right but rather experienced and used as instruments. In our clinical experience, therapists (and others) are most valued if they maintain an emotional distance and refrain from empathic interventions. This was seen in the example quoted above of the patient who said: “When you are so kind to me, I want to hit you!”

The need for personal gain can easily be misunderstood: the benefit is found in the enhancement of the subjective self. The instrumentality of relationships is a defense against the unbearable feeling of being dependent on the relationship (Kernberg, 1975, 1984). The exploitative quality of relations looks superficially like a “gain” but as therapists we should not forget that this gain involves a price: the patient lacks the capacity for self-soothing and existential loneness results. Characteristically, others are usually idealized or devaluated excessively and inappropriately. The patient may hyper-idealize others in order to comfortably warm him- or herself in the heat of their radiance: “Look how great we are!” (“mirror transference,” Kohut, 1972). Hyper-idealizing someone also places the patient in the position of being the one who has the expertise to judge, which fuels feelings of superiority. Excessive devaluation comes to the fore if the existence of the other threatens the stability of the subjective self by association: “Who am I, if I am associated with that loser?” A patient said to one of us: “Are you divorced? Because if you are, how can you help me with my relational problems when you can't handle them yourself?” The often bitter and aggressive nature of devaluation serves to enhance the subjective self. Idealization and devaluation are associated with an insecure dismissing-avoidant attachment style (Tolmacz and Mikulincer, 2011). Ambivalence is seldom cherished as a valuable state of mind; instead, relations are about winning or losing, and jealousy is omni-present.

Anything with relational implications will be dismissed if it might give pleasure and make one emotionally alive. The evaluation of anniversary gifts is exemplary: a patient with grandiose narcissism said: “Getting presents for my anniversary is only a means of bringing more worthless trash into my house.” His vulnerable counterpart always bought himself a present after his birthday, shielding himself from the disappointment that others may not give him the “right” presents. Describing the basic relational patterns of patients with NPD, Lachkar (2008) writes that their partners are quite often diagnosed with BPD. It is a tale of the deaf leading the blind and, usually, the relationship falters when the partner with BPD matures and becomes less dependent and anxious.

Sexuality in relationships is often complicated. The patient tries to avoid the humiliation of having to display needs and wishes, and of experiencing vulnerability: “Hell is other people,” said Sartre (1943). Psychoanalyst Green adds to Sartre's dictum: “Hell is not other people, but rather the body. … The body is a limitation, a servitude. … The body is his absolute master–his shame” (Green, 1997, p. 127). Sexuality is often reduced to a mere physical pleasure, whether or not permeated with fantasies of being the greatest lover. Extreme self-centeredness or other-centeredness during lovemaking is characteristic, as reciprocity and empathic attunement are avoided. The partner is treated instrumentally: “What value does the other's sexual pleasure have for myself as a lover?” A male patient broke up his marriage after discovering he had been lied to for years: with great shame, his wife had told him she was unorgiastic and had faked orgasms. His self-worth as a great lover crumbled.

Sexuality can turn into perverse love: sexual excitement becomes the substitute for love and the longing of the other serves to strengthen the cohesion in the self. The own body, the other's body, or a fetish becomes a sexual object, an eroticized self which is constantly longing for stimulation (Akhtar, 2009). It is not uncommon to find NPD patients who also suffer from hypochondria: the frail implicit self has developed alongside a frail bodily self.

## Reflection on the Narcissistic Personality Traits of Grandiosity and Attention Seeking

It should be remembered that the AMPD characterizes each personality disorder on the basis of a specific pattern of personality dysfunctions and traits. In the section above, we described the patterns of this pattern in NPD by looking at a unique pattern of self-impairments, which are evaluated by focusing on identity and self-direction, and of interpersonal functioning, which is evaluated by focusing on empathy and intimacy. We now turn to the unique trait profile of NPD: grandiosity and attention seeking.

### Evaluating Personality Traits: Grandiosity

The AMPD conceptualizes grandiosity as “Feelings of entitlement, either overt or covert; self-centeredness, firmly holding to the belief that one is better than others; condescension toward others” (American Psychiatric Association, 2013, p. 768).

The description of feelings of entitlement, either overt or covert, fits in well with Pincus and Lukowitsky's (2010) suggestion that grandiose and vulnerable narcissism can be expressed both overtly and covertly and, consequently, that feelings of entitlement should not only be associated with grandiose narcissism. This perspective confirms our clinical experience but it is, at the same time, subject to some theoretical discussion. The first edition of the Psychodynamic Diagnostic Manual (PDM; PDM Task Force, 2006) differentiated between an arrogant/entitled and a depressed/depleted subtype of narcissism (Blatt, 1974). The PDM characterized “depleted self-imagery, angry, shameful, and depressed affects, self-criticism and suicidality, and interpersonal hypersensitivity/social withdrawal” (Morey and Stagner, 2012, p. 910). In the PDM-2, which focuses on personality styles and not on personality disorders, entitlement is mentioned only as a pattern in adolescents with narcissism (Lingiardi and McWilliams, 2017).

The same applies to clinging to the belief that one is better than others and condescension toward others. These characteristics can also be seen in both expressions of narcissism, and particularly in masochistic narcissism: the grandiosity of suffering is hidden by silently and secretly experiencing the grandiosity of being able to bear any adverse events (Fairbairn, 1940; Kernberg, 2007).

Entitlement and condescension are two characteristics of narcissism that have given narcissism its negative connotation in everyday speech. In psychodynamic theory, there is a close association between the nature of entitlement and a defensive wilful resistance to dependency and reciprocity. Patients wilfully decline to relate with another in order to get what they want; instead, they expect it to be served or granted without having to ask explicitly. Asking is about losing, as asking would acknowledge neediness and dependency. Research has shown that excessive and restricted forms of relational entitlement are significantly associated with insecure attachment styles (Tolmacz and Mikulincer, 2011). In the clinical situation, we encounter patients who literally refuse to give up their entitlement. Their narcissistic rage is fuelled to no purpose by a feeling of entitlement and by the demand to be compensated for the misdeeds or shortcomings of persons or circumstances in the past. In our consulting room, we meet patients who cannot cut their losses with respect to situations in the past and, in their hate, remain attached to a parent in an obsessive and spiteful way. Working through this persistence is often painstakingly difficult because the rage prevents patients from establishing the psychological distance through the self-as-object that is necessary to see the insanity of their expectations.

### Evaluating Personality Traits: Attention Seeking

The AMPD conceptualizes attention seeking as: “Excessive attempts to attract and be the focus of the attention of others; admiration seeking” (American Psychiatric Association, 2013, p. 768).

Again, it is easy to associate these criteria with overt narcissism and therefore fail to notice covert attention-seeking involving putting others in the spotlight. The essence of this latter type of self-esteem regulation is that patients subconsciously see their self-effacing behavior in the service of the well-being of others as support for their self-esteem. However—and this is essential—the relationship with the other is instrumental and can therefore be perceived by the other as manipulative. In intersubjective terms: the other is treated as an object that possesses conditional value. Even when the other is placed explicitly in the spotlight and patients do not get any exposure for themselves, the self-esteem of vulnerable patients may be enhanced considerably as they attribute the other's greatness to their own contribution (Kohut's “narcissistic mirroring needs”). Vulnerable narcissism is often found in persons who claim to function best as “the second person.”

Attention seeking therefore involves not only seeking admiration for oneself directly; it also includes forms of behavior in which admiration is given to others. This is a classic pitfall in treatment when, in the transference-countertransference matrix, the patient and therapist build up a mutual admiring collusion as both being “the best ever, together.” This form of covert, “eager to please,” narcissism is well-documented in psychoanalytic literature but often underdiagnosed in clinical practice. “Eager to please” narcissism is often associated with parentification in childhood (Miller, 1981).

## Concluding Remarks

In this article we integrated Pincus and Lukowitsky's (2010) hierarchical model of pathological narcissism, contemporary psychodynamic concepts of narcissism, and the diagnostic concept of narcissism in the AMPD.

Pincus and Lukowitsky encourage clinicians to use this hierarchical model as it opens up opportunities for shared points of interest in empirical research from different scholarly perspectives. Capacities for self-regulation and emotion regulation can, for example, be operationalized from social-learning theory and from a psychodynamic perspective, with each adding valuable knowledge. Pincus and Lukowitsky's valuable review showed there has been hardly any research into NPD with a clinical patient sample. More research involving a clinical sample is therefore needed. In addition, researchers could adapt their methods in order to conduct research that is clinically relevant for mental health care by focusing on phenomena that can be addressed in psychotherapeutic treatment. Pincus and Lukowitsky's review also showed that narcissism research is skewed by the use of the Narcissistic Personality Inventory, which mostly assesses adaptive expressions of grandiose narcissism. In the hierarchical model, vulnerable narcissism emerges as a relatively new concept for non-psychodynamically informed researchers and therapists, and additional measures have to be developed to cover this concept.

For us, one of the major advantages of the AMPD is the use of structured clinical evaluations of disturbances of the self and interpersonal functioning. In the present paper, we have discussed at length the thematic content of the AMPD. As psychodynamically oriented therapists, we are enthusiastic about the opportunities to include psychodynamic and structural concepts (see also: Bornstein, 2015). In addition to the thematic content, we welcome the dimensional evaluation of the severity of personality disorder pathology, as operationalized in DSM AMPD Criterion A, which can be assessed by instruments like the Semi-structured Interview for Personality Functioning (STiP-5.1) and Level of Personality Functioning Scale Self-Report (LPFS-SR) (Hutsebaut et al., 2017), or scorings based on the Object Relations Inventory (ORI) (Borroni et al., 2020).

In addition to the thematic content, we welcome the dimensional evaluation of the severity of personality disorder pathology. Kernberg's structural model for personality organization could provide an insight into the severity of all these thematic elements, in other words whether relevant psychodynamic features are organized in a neurotic or high-level/low-level borderline way. This provides the practitioner with information about the prognosis and the indication for the treatment model (Caligor and Stern, 2020).

We also acknowledge that there are a number of discussion points. Following the example of all psychodynamic theories, the AMPD assumes in the case of NPD that there is a disturbance that goes back to early child development. However, in all honesty, there is still no empirically derived theory for the etiology of grandiose and vulnerable narcissism, even though there is now more research with children from researchers like Brummelman et al. (2016). Relational psychodynamic theory has undeniably been supplemented with clinical child research into attachment, mentalization, emotion regulation, and parenting styles. It is, however, unfortunate that research has also shown that the link between childhood experiences and later emotional disturbances is relatively weak. More empirical data about attachment styles and emotion regulation styles in patients with narcissistic pathology would be welcome as support for the unique pattern of narcissistic relational dynamics.

In the final evaluation of the four AMPD DSM-5 elements of personality functioning, all the elements seem to have equal importance but clinical experience and psychodynamic clinical theory clearly place most emphasis on the element of identity, with self-regulation and emotion regulation as the most important aspect of this element. This problem can be resolved by further research into the relative importance of the four elements of personality dysfunction. The need to evaluate the severity of impairment in personality functioning is a valuable element in the proposed diagnostic criteria for NPD that psychodynamically oriented therapists could use to their benefit. We believe that the criteria for the two personality traits, grandiosity and attention seeking, rely too heavily on the definition of NPD in the traditional DSM-5, with its focus on grandiose narcissism. However, further research could determine whether only these two traits pertain to NPD or if other traits might be relevant as well. Future research using the Level of Personality Functioning Scale, as proposed in the AMPD, will provide ample opportunities for introducing a more sophisticated psychodynamic perspective.

The AMPD comes close to how psychoanalytic therapists could conceptualize their daily practice (see also: Caligor and Stern, 2020). As mentioned here, a positive aspect of the AMPD is that the diagnostic evaluation of the level of personality functioning is based on a structured clinical evaluation of four clinically relevant elements. The model addresses all the theoretical and clinical elements of pathological narcissism mentioned, such as self-regulation, affect regulation, interpersonal difficulties, grandiose/vulnerable, and covert/overt. In contrast to DSM-5 personality disorders in Section II, the AMPD clearly offers a more integrative approach. However, understandably, the basic tenet in clinical theory that distancing from the significant other forms the basis for developing NPD is not operationalized in the AMPD. Ultimately, this distancing can only be clinically inferred by assessing its consequences, which are described in the AMPD.

Now, after all this theory, the proof of the pudding is once again in the eating. In our case, the proof is to be found in the therapies we provide. Many guidelines for treating pathological narcissism have been developed in the last 10 years. Choi-Kain (2020) advocates using General Psychiatric Management, while others propose modifications of existing evidence-based treatment models for BPD to treat pathological narcissism: Mentalization-Based Treatment (Drozek and Unruh, 2020), Transference Focused Psychotherapy (Diamond and Hersh, 2020), Dialectical Behavior Therapy (Reed-Knight and Fischer, 2011), or Schema Focused Therapy (Young et al., 2003). Nevertheless, others focus on specific themes when treating pathological narcissism, for example in psychodynamic therapy (Crisp and Gabbard, 2020) or the client-centered Clarification-Oriented Psychotherapy (Maillard et al., 2020). Traditional high-frequency psychoanalysis—three to five weekly sessions on the couch—seems to have missed the boat in terms of establishing a position in the discussion.

After we concluded the draft version of this publication, the paper *The “Why” and “How” of narcissism. A process model* (Grapsas et al., 2020) came to our attention. It comes from the field of social learning and experimental psychology. Almost none of the references in that paper overlap with those in the present paper. Given the realization that there are so many overlaps, it is shocking that we seem to know so little about each other's work. For example, both fields look at internal processing in subjects with narcissism. Grapsas et al. (2020) propose a self-regulation model of grandiose narcissism that illustrates an interconnected set of processes through which narcissists pursue social status in their moment-by-moment transactions with their environments. In the same way, Ronningstam (2020b) draws attention to internal processing in patients and how it contributes to narcissistic personality functioning. “Studies provide evidence for a neuropsychological core deficit in individuals with pathological narcissism or NPD, which affects their ability to access, tolerate, identify, and verbalize emotions” (Ronningstam, 2020b, p. 85). Narcissism seems to be associated with many bioneurological phenomena that are prototypical for narcissism. Experimental research has found increased sensitivity to subtle cues of non-acceptance in facial expressions, the “denial” of physical shame reactions after being devalued, the rise of cortisol levels in situations of social threat, or the activation of brain regions sensitive to pain in response to exclusion. Ronningstam argues that more attention should be paid to all kinds of internal processing from a neuropsychoanalytic point of view. As in the treatment of traumatized patients, this approach could inform the therapist in therapeutic stalemates.

Affective neuroscience can enlighten the neurological correlates of our subjective states. Solms (2017) argues that striving for homeostasis of the self pertains specifically to “basic (brainstem) consciousness, which consists in *states* rather than *images*” (Solms, 2017, p. 6). This is the self-system Schore calls the implicit self, associated with the unrepressed unconscious. Central to Schore's thinking is the notion that the idea of a single unitary self is misleading: “What we call the self is in reality a system of self states, that develop in the early years, but grow to more complexity during the life span” (Schore, 2017, p. 74). In the first year of life, the structuralization of the right brain self develops in the course of the interdependent interaction between child and caretakers (*self-objects*), especially through processes of mismatch and repair in attachment, and with it (mal)adaptive implicit self-regulation processes develop. In early development, this implicit self, supposedly located in the lateralized right brain, is basically relational, as the self-states develop out of the interaction with the self-objects. Schore (2009, 2017) locates the brain's major self-regulatory systems in the orbital prefrontal areas of the right hemisphere. Its functioning belongs to the unrepressed unconscious; its content can be felt but cannot be translated into words or symbols. Accordingly, in psychotherapy, it cannot be reached through interpretations making the unconscious conscious, but it becomes visible in enactments between psychoanalyst and patient. Somewhat later in early development, after the second year, the verbal, conscious left lateralized self-system (“left mind”) develops. Schore writes: “Despite the designation of the verbal left hemisphere as “dominant” due to its capacities for explicitly processing language functions, it is the right hemisphere and its implicit homeostatic survival and affect regulation functions that are truly dominant in human existence” (Schore, 2017, p. 74).

The central challenge in the decade to come would seem to be to differentiate between NPD from BPD and to establish specific recommendations for treatment. Indeed, we agree with the comment made by Choi-Kain (2020) that was quoted in the introduction of this paper, that we can now look ahead to a new wave of investigation and treatment development.

## Author Contributions

All authors listed have made a substantial, direct and intellectual contribution to the work, and approved it for publication.

## Conflict of Interest

The authors declare that the research was conducted in the absence of any commercial or financial relationships that could be construed as a potential conflict of interest.

## Footnotes

^1^This research outcome has been reframed by us, as Tanzilli and Gualco use different subtypes of narcissism.
